# Investigating Pericarditis Diagnosis in a Tertiary Pericardial Center: A Descriptive Study

**DOI:** 10.1016/j.jacadv.2025.102335

**Published:** 2025-11-18

**Authors:** Abdel Hadi El Hajjar, Ziad Zalaquett, Akiva Rosenzveig, Alveena Batool Syed, Astefanos Al-Dalakta, Zoha Majeed, Michael Villalonga, Jibran Ikram, Muhammad Ehsan, Issam Motairek, Mary Heine, Felix Berglund, Tom Kai Ming Wang, Allan Klein

**Affiliations:** Center for the Diagnosis and Treatment of Pericardial Diseases, Section of Cardiovascular Imaging, Department of Cardiovascular Medicine, Heart, Vascular, and Thoracic Institute, Cleveland Clinic, Cleveland, Ohio, USA

**Keywords:** LGE-MRI, long-COVID, misdiagnosis, pericarditis

## Abstract

**Background:**

Pericarditis diagnosis can be challenging due to its nonspecific presentation, which may lead to frequent misdiagnosis.

**Objectives:**

In this study, the authors aimed to report the rate of misdiagnosed pericarditis and describe the characteristics of these patients.

**Methods:**

Between September 2022 and September 2023, patients referred to the Pericardial Center with a diagnosis of pericarditis were enrolled in a prospective registry. Data were collected using a standardized history and physical examination template. Diagnosis was established according to the 2015 European Society of Cardiology guidelines. The prevalence of misdiagnosed pericarditis was calculated. Demographics, comorbidities, symptoms, laboratory results, and imaging were compared between the accurately diagnosed (group 1) and misdiagnosed pericarditis cohorts (group 2).

**Results:**

A total of 170 patients were included; the mean age was 49.1 years. Thirty-five percent of patients were misdiagnosed. Demographics were similar in both groups. Significant differences were found between group 1 and group 2) in coronary artery disease (17.3% vs 5.0%, *P* = 0.02), valvular disease (23.6% vs 8.3%, *P* = 0.01), and atrial fibrillation (24.5% vs 6.7%, *P* < 0.01). Significant late gadolinium enhancement on magnetic resonance imaging, raised inflammatory markers, electrocardiogram changes, and specific pericardial pain were more common in the accurately diagnosed group (*P* < 0.05). COVID-19 infection was significantly higher in the misdiagnosed group (16.7% vs 6.4%, *P* < 0.05), whereas cardiac surgery was significantly lower (3.3% vs 18.2%, *P* < 0.05).

**Conclusions:**

Thirty-five percent of patients referred to the pericardial center are misdiagnosed with pericarditis. Inflammatory markers and late gadolinium enhancement on magnetic resonance imaging can help refine the diagnosis. COVID-19 infection is associated with higher rates of misdiagnosis.

Pericarditis is the inflammation of the pericardium that varies significantly in presentation, symptoms, and prognosis, making its diagnosis challenging.^1^ Timely and accurate diagnosis is critical to initiating appropriate treatment and preventing life-threatening complications.^2^ However, although pericarditis can often be distinguished from other differentials based on history alone, atypical presentations and overlapping symptoms in referred patients can complicate the diagnosis, leading to potential misdiagnosis.

The Cleveland Clinic Pericardial Diseases Center is a multidisciplinary specialty treatment group dedicated to diagnosing and treating pericardial diseases. Many patients are being referred for pericarditis management. In recent years, a thorough review of the patient’s history and physical examination has led to many diagnoses other than pericarditis. This trend points to the potential overutilization of health care resources due to misdiagnosis. Given its implication, it is essential to explore the prevalence of misdiagnosed pericarditis and understand the factors contributing to this phenomenon.

This study aims to determine the incidence of misdiagnosed pericarditis among patients referred to a tertiary care center and to characterize the clinical and diagnostic findings within this population.

## Materials and methods

### Study population and study design

The study focuses on a prospective cohort of individuals who presented to the Cleveland Clinic Pericardial Center with a diagnosis of pericarditis. It was conducted between September 2022 and September 2023. Exclusion criteria included patients <18 years old, patients referred to the pericardial center for a different disease, or patients previously diagnosed with pericarditis by a physician at the Cleveland Clinic Pericardial Center.

**Figure 1:**
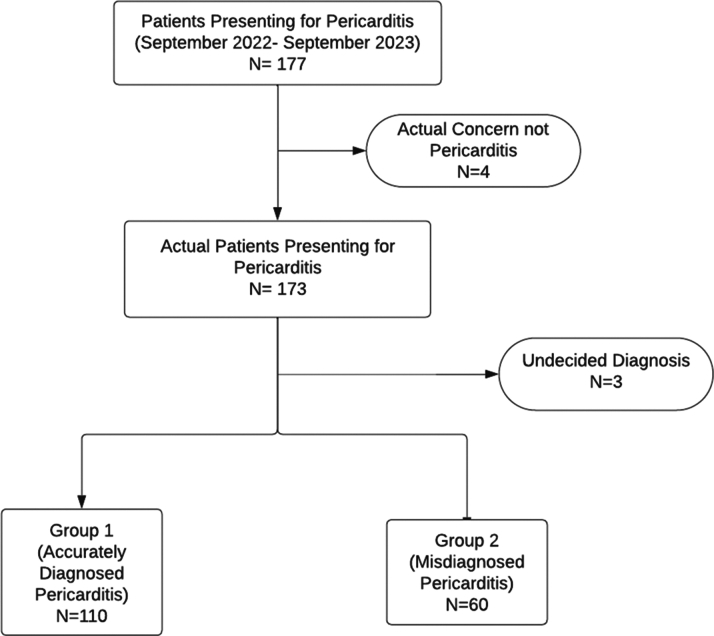
Study Design

Information regarding all patients is documented in a standardized “history and physical” format across all providers. Pericarditis diagnosis was established according to European Society of Cardiology (ESC) 2015 guidelines at the pericardial center after 2 physicians reviewed the history, labs, and imaging. The prevalence of misdiagnosed pericarditis was determined accordingly. Patients who were accurately diagnosed with pericarditis will be referred to as group 1, and patients who were misdiagnosed will be referred to as group 2. Characteristics of patients were compared in both groups. The design of the study is summarized in Figure 1.

### Study variables

Baseline patient characteristics, including demographics, comorbidities, medication use, and laboratory data, were obtained from the electronic medical records. Most data were extracted from the standardized “history and physical” template for all new patients at the pericardial center. The template included clinical characteristics such as chest pain, risk factors, prior medical and surgical history, smoking status, physical activity, and physical activity levels. Laboratory, imaging, and electrocardiogram (EKG) findings were also extracted from the template. A chart review was performed to find missing data from the template. All characteristics were collected and stored in a Health Insurance Portability and Accountability Act (HIPAA)-compliant, secure database for analysis. The Cleveland Clinic Institutional Review Board approved this study.

### Outcome definitions

Our study used the 2015 ESC diagnostic criteria for pericarditis^1^ as the gold standard. Patients are considered accurately diagnosed (group 1) with pericarditis if they have 2 out of 4 symptoms: pericardial chest pain, EKG changes suggestive of pericarditis, the presence of pericardial effusion, or pericardial rub. Pericarditis is called incessant if it lasts between 4 weeks and 3 months, chronic if it lasts more than 3 months, and recurrent if another episode of pericarditis recurs after a symptom-free interval of 4 to 6 weeks. Patients with all types of pericarditis are included in group 1. Patients who fail to meet those criteria are classified as misdiagnosed pericarditis and therefore included in group 2. Long-COVID was diagnosed at the discretion of the treating clinician, based on persistent or new-onset symptoms lasting ≥4 weeks after confirmed SARS-CoV-2 infection.

### Statistical analysis

All statistical analyses were performed using SPSS Statistics (version 27.0.1; IBM Corp). Continuous variables were reported as mean ± SD. Categorical variables were reported as frequencies and percentages. Proportions presented with 95% CIs were calculated using the Wald method. Depending on the normality of the distribution evaluated by the Shapiro-Wilk test, the means of continuous variables between the 2 groups were compared using the t-test or the Mann-Whitney test. A chi-square or Fisher exact test were performed to compare the percentages of categorical variables, as appropriate.

## Results

### Prevalence of misdiagnosed pericarditis

A total of 177 patients presented to the pericardial center as a referral for newly diagnosed pericarditis between September 2022 and September 2023. Four patients were excluded as their presenting concern was not related to pericarditis. Three other patients were excluded as the diagnosis was ambiguous and remained undecided after evaluation. A total of 170 patients were included in the analysis. Forty-one percent of the patients were self-referred, whereas 46% were referred by another physician, all of whom were cardiologists. Thirteen percent were referred following a hospital admission. Of 170 patients, 110 were accurately diagnosed with pericarditis, and 60 were misdiagnosed with pericarditis. The prevalence of misdiagnosed pericarditis was 35%.

### Baseline demographics and comorbidities

The mean age of the study population was 49.1 years (95% CI: 46.6-51.6), and 53% were female (95% CI: 45.4%-60.5%). There was no significant demographic difference between the 2 groups (Table 1). The mean age was 51.4 in group 1 vs 45.0 years in group 2 (*P* = 0.90), and the proportion of females was 49% vs 60% (*P* = 0.17). Athletes accounted for 14% of the accurately diagnosed group and 21% of the misdiagnosed group (*P* = 0.29). Racial distribution was similar in both groups: 86% vs 91% White and 6% vs 9% Black in the accurate and misdiagnosed group, respectively (*P* = 0.14). Patients in the accurately diagnosed group had significantly higher rates of cardiac comorbidities, including atrial fibrillation (AF) (25% vs 7%, *P* < 0.01), coronary artery disease (CAD) (17% vs 5%, *P* = 0.02), and valvular disease (24% vs 8%, *P* = 0.01). No significant difference was found between groups regarding the prevalence of heart failure with reduced ejection fraction (*P* = 0.82) and preserved ejection fraction (*P* = 0.88). There was no significant difference in other comorbidities between the 2 groups. The data are further detailed in Table 1.

**Table 1:** Comparison of Baseline Demographics and Comorbidities Between Misdiagnosed and Accurately Diagnosed Pericarditis Patients

	Accurately Diagnosed Pericarditis (Group 1)	Misdiagnosed Pericarditis (Group 2)	*P* Value
Baseline demographics			
Age (y)	51.4 ± 16.4	45 ± 16.1	0.90
Body mass index (BMI) (kg/m^2^)	29.2 ± 6.9	25.7 ± 6.5	0.80
Sex (%)			
M	56 (51%)	23 (38%)	0.17
F	54 (49%)	37 (62%)	
Race (%)			
White	91 (83%)	50 (83%)	0.27
Black	6 (5%)	5 (8%)	
Asian	4 (4%)	0 (0%)	
Multi	6 (5%)	2 (3%)	
Smoker (%)			
Current	6 (5%)	4 (7%)	0.91
Former	22 (20%)	10 (17%)	
Athlete (%)	15 (14%)	12 (20%)	0.53
Pain characteristics, median (IQR)			
Total number of episodes of pericarditis	2 (IQR 1-4)	1 (IQR 0-2.5)	0.29
Number of emergency room visits	2 (IQR 1-3)	0 (IQR 0-2)	**<0.01**
Number of hospitalizations for pericarditis	1 (IQR 0.5-2)	0 (IQR 0-1)	**<0.01**
Pain score (/10)	7 (IQR 5-9)	6 (IQR 4-8)	0.05
Type of pericarditis (%)			
Acute	8 (7%)	13 (22%)	0.06
Chronic	17 (15%)	14 (23%)	
Recurrent	77 (70%)	31 (52%)	
Incessant	8 (7%)	2 (3%)	
Medication used during episodes of pericarditis (%)			
NSAID	78 (71%)	32 (53%)	**0.02**
Colchicine	93 (85%)	46 (77%)	0.21
Glucocorticoids	51 (46%)	10 (17%)	**<0.01**
IL-1 inhibitors (anakinra, rilonacept, canakinumab)	20 (18%)	4 (7%)	0.06
Comorbidities (%)			
Atrial fibrillation	27 (25%)	4 (7%)	**<0.01**
Cancer	5 (5%)	5 (8%)	0.32
Coronary artery disease	19 (17%)	3 (5%)	**0.03**
Diabetes	10 (9%)	3 (5%)	0.55
History of stroke	7 (6%)	0 (0%)	0.05
Heart failure with preserved EF	8 (7%)	4 (7%)	0.88
Heart failure with reduced EF	3 (3%)	2 (3%)	0.82
Hyperlipidemia	31 (28%)	11 (18%)	0.16
Hypertension	38 (35%)	15 (25%)	0.20
Kidney disease	8 (7%)	2 (3%)	0.50
Obstructive sleep apnea	13 (12%)	4 (7%)	0.29
Rheumatologic disease	14 (13%)	12 (20%)	0.22
Valvular disease	26 (24%)	5 (8%)	**0.01**

Bold values indicate statistical significance at *P* < 0.05. EF = ejection fraction; IL = interleukin; NSAID = nonsteroidal anti-inflammatory drug.

### Pericarditis characteristics

Of all the patients, 10.6% had acute pericarditis, 18.2% had chronic pericarditis, 65.2% had recurrent pericarditis, and 6.1% had incessant pericarditis. There was no significant difference in pericarditis type between the 2 groups. There was also no significant difference in pain score and number of chest pain episodes in both groups. Group 1 patients had a higher number of emergency room visits vs misdiagnosed pericarditis (2 vs 1, *P* < 0.01) and a higher number of hospitalizations (1.4 vs 0.5, *P* < 0.01).

### Precipitating factors

**Figure 2:**
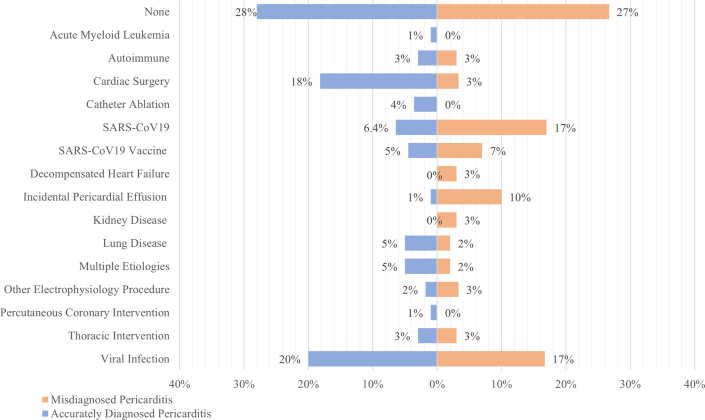
Comparison of Precipitating Factors Between Misdiagnosed and Accurately Diagnosed Pericarditis Patients

There was a significant difference in precipitating factors that preceded symptoms of pericarditis diagnosis in both groups (*P* < 0.05). Eighteen percent of patients with accurately diagnosed pericarditis had cardiac surgery before symptom onset vs 3% in misdiagnosed patients. Catheter ablation (4% vs 0%) and percutaneous coronary intervention (1% vs0%) before symptom onset were more frequent in group 1. On the other hand, misdiagnosed patients had higher rates of incidental pericardial effusion (1% vs 10%) and COVID-19 infection (6% vs 17%). Another viral infection preceded symptoms in 20% of patients in the accurately diagnosed group vs 17% in the misdiagnosed group. No precipitating factors were identified in 28% and 27% of patients in groups 1 and 2. All precipitating factors are shown in Figure 2.

### Diagnostic markers

**Figure 3:**
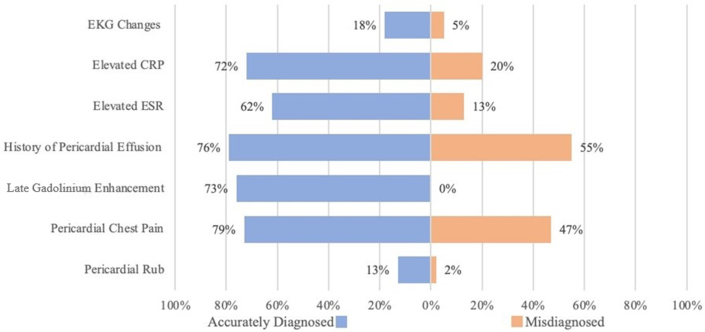
**Comparison of Diagnostic Criteria Between Misdiagnosed and Accurately Diagnosed Pericarditis Patients** CRP = C-reactive protein; EKG = electrocardiogram; ESR = erythrocyte sedimentation rate; MRI = magnetic resonance imaging.

There was no significant difference in pericardial effusion in groups 1 and 2 (28% and 55%, respectively, *P* = 0.25). All other markers were significantly higher in group 1: history of pericardial effusion (79% vs 55%, *P* < 0.01), EKG changes (18% vs 5%, *P* = 0.02), elevated C-reactive protein (71% vs 20%, *P* < 0.01), elevated erythrocyte sedimentation rate (62% vs 13%, *P* < 0.01), pericardial chest pain (*P* < 0.01), and pericardial rub (*P* = 0.01). Seventy-six percent in group 1 had pericardial enhancement on late gadolinium enhancement magnetic resonance imaging (LGE-MRI). In contrast, none of the misdiagnosed patients had pericardial enhancement (*P* < 0.01). Diagnostic markers are shown in Figure 3.

After review of the 2015 ESC criteria in the misdiagnosed group, we found that 7 patients (11.7%) had zero criteria, 38 (63.3%) had one criterion, 14 (23.3%) had 2 criteria, 1 (1.7%) had 3 criteria, and none had all 4 criteria. The ESC criteria counts reflect the referring diagnosis; on expert reassessment at our center, some of these criteria did not meet true diagnostic thresholds.

### Alternative diagnoses in patients with misdiagnosed pericarditis

**Figure 4:**
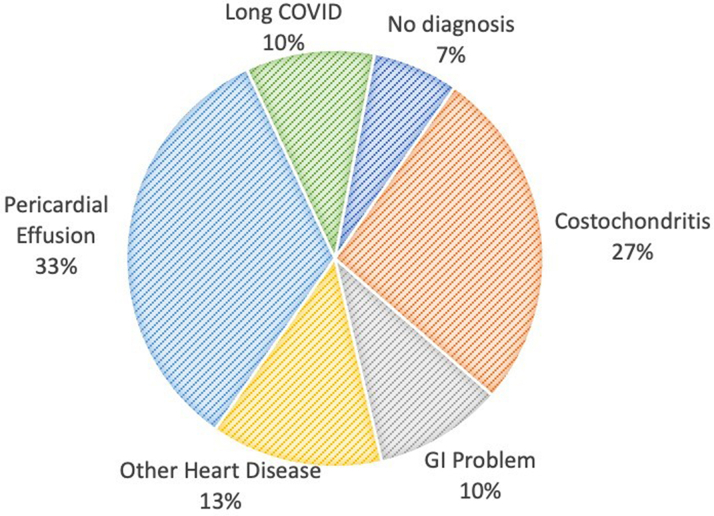
Alternative Diagnoses Established in Patients With Misdiagnosed Pericarditis. GI = gastrointestinal.

The charts of the patients with misdiagnosed pericarditis were reviewed to determine if an alternative diagnosis was eventually made (Figure 4). Incidental findings of pericardial effusion (33%) and costochondritis (27%) were the most common alternative diagnoses. Other diagnoses included other cardiovascular problems (13%), including decompensated heart failure, gastrointestinal symptoms (10%), including acid reflux and ulcers, and long COVID (10%).

**Central Illustration:**
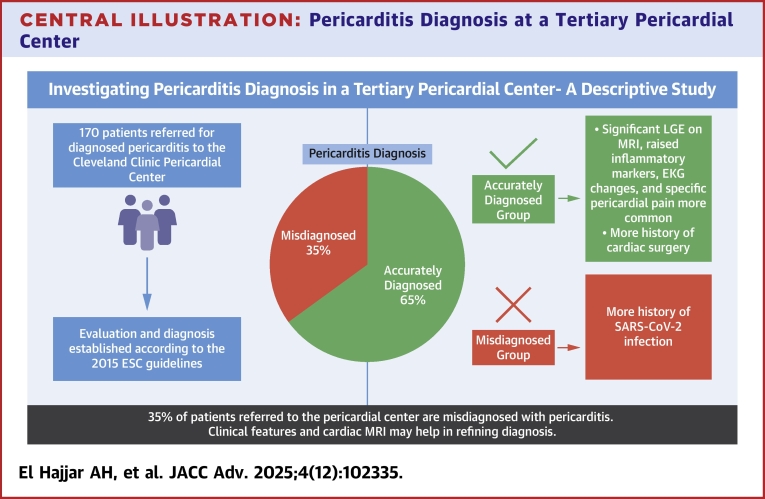
**Pericarditis Diagnosis at a Tertiary Pericardial Center** Pericarditis was misdiagnosed in 35% of cases referred to a tertiary pericardial center, where the evaluation and diagnosis were made according to the gold standard 2015 ESC guidelines. MRI enhancement, inflammatory markers, EKG changes, typical pericardial chest pain, and a history of cardiac surgery were more common in the accurately diagnosed group, while a history of SARS-CoV-2 was more common in the misdiagnosed group. Clinical history and objective measures like LGE on MRI may therefore help in refining pericarditis diagnosis. EKG = electrocardiogram; ESC = European Society of Cardiology; LGE = late gadolinium enhancement; MRI = magnetic resonance imaging.

## Discussion

Our study found that the rate of misdiagnosed pericarditis is 35%. Patients with misdiagnosed pericarditis had fewer cardiac comorbidities and were unlikely to be preceded by a cardiac intervention such as catheter ablation or cardiac surgery. COVID-19 infection and incidental findings of pericardial effusion were related to misdiagnosis. Pericardial enhancement on MRI and raised inflammatory markers were lower in the misdiagnosed group (Central Illustration).

This is the first study to report the rate of misdiagnosed pericarditis. The high rate could be partly attributed to wrong clinical judgment and failure to adhere to the 2015 ESC criteria, the gold standard for diagnosis. One of the significant factors driving misdiagnosis is the subjective description of chest pain. In misdiagnosed patients, 47% had atypical and non-classic pericardial chest pain. Typical pericardial pain is sharp and pleuritic and improves by leaning forward.^1^ This highlights the importance of accurate history-taking while establishing the diagnosis. Another critical aspect of history-taking is the presence of pericardial effusion. Many patients in our study were diagnosed with pericarditis for having an incidental pericardial effusion alone. This sheds light on the spectrum of pericardial disease. Pericardial effusion is 1 of 4 criteria to help diagnose, however the presence of pericardial effusion without pericardial chest pain does not necessarily mean the patient has pericarditis.

Implications of such misdiagnosis include the inappropriate use of steroids, biologics, or other treatments, leading to unnecessary exposure and potential adverse effects. Patients with recurrent pericarditis may be considered for biologic therapies such as rilonacept, anakinra or even pericardiectomy. For instance, 1 of the patients included in the study was referred for recurrent pericarditis and was already on biologics. On our evaluation, the patient did not meet the criteria for pericarditis and was labeled as misdiagnosed. Further workup revealed spinal stenosis as the actual cause of her symptoms, and her pain improved with neurosurgical intervention, and she was eventually weaned off rilonacept. This highlights the importance of diagnostic precision in guiding management.

In addition, our study showed that although most comorbidities are unrelated to misdiagnosis with misdiagnosed patients had fewer incidences of cardiac comorbidities (CAD, AF, and valvular heart disease). After chart review, it is most likely that patients with more comorbidities tend to have more cardiac interventions and tend to develop postcardiac injury syndrome (PCIS). PCIS is a well-documented and commonly encountered syndrome in patients with pericarditis. PCIS consists of a heterogeneous group of inflammatory conditions of the pericardium, epicardium, or myocardium. PCIS has been noted as one of the most common etiologies of pericarditis^3^ and is subclassified into postmyocardial infarction (MI) pericarditis, postpericardiotomy syndrome, and post-traumatic pericarditis.^4^ Postpericardiotomy syndrome occurs in 10% to 40% of patients undergoing cardiac operations^1^ and is the most common etiology of PCIS.^5^ This is consistent with the findings of our study, as a high percentage of patients with accurately diagnosed pericarditis had cardiac surgery before pericarditis onset, with 70% of them having an inflammatory phenotype, a pattern that aligns with postpericardiotomy syndrome. Electrophysiology procedures such as AF ablation constitute a substantive percentage of patients with PCIS, with PCIS complications affecting 3.7% to 10% of ablation procedures.^6^^,^^7^ In our study, many accurately diagnosed cases of pericarditis had a catheter ablation before the symptom onset. PCIS has also been well described in valvular surgeries, most commonly in aortic and mitral valvular procedures,^8^^,^^9^ and thus, the higher rate of valvular disease in patients with an accurate diagnosis. We also found a correlation between patients accurately diagnosed with pericarditis and those with CAD. Although this study does not delve into the specific CAD pathology that is antecedent to the development of pericarditis, Thadani et al^10^ describe a significant relationship between MI and the development of post-MI pericarditis. Figueras et al^11^ expand on this further, suggesting that patients with a first anterior MI had a higher propensity of developing pericardial effusion with possible development of tamponade physiology. Verma et al^12^ shed light on the multiple sequelae of acute MI, including late-presenting pericarditis (also known as Dressler syndrome), which makes it a challenging diagnosis. This requires a high index of clinical suspicion and an accurate history.^13^ Imazio et al^14^ expand on this, shedding light on the increasing prevalence of pericardial disease in late-presenting MI. This suggests that the presence of cardiac injury before symptom onset might be in favor of accurately diagnosing pericarditis.

Another significant finding in our study is that patients with misdiagnosed pericarditis had a higher prevalence of COVID-19 infection as a precipitating factor. Some reports in the literature suggested that pericarditis is sometimes underdiagnosed after COVID-19^15^ or as part of PCIS.^16^ It has been shown previously that COVID-19 is associated with an increased risk of pericarditis. SARS-CoV-2, the virus responsible for COVID-19, enters cells by binding to the angiotensin-converting enzyme 2 receptor, which is expressed in various tissues, including the heart, and requires the assistance of transmembrane protease, serine 2 for cell entry.^17^ This viral entry can lead to direct cardiac damage, resulting in perimyocarditis, with additional contributions from the body's immune response, including a cytokine storm that may cause further injury even when viral replication in the heart is minimal.^18^ Before the COVID-19 pandemic, the incidence of acute pericarditis was relatively low, estimated at 3.3 cases per 100,000 individuals.^19^ However, during the pandemic, the incidence of myocarditis and pericarditis associated with SARS-CoV-2 infection increased significantly, with some studies estimating an incidence as high as 150 cases per 100,000 individuals.^20^

In addition, myocarditis and pericarditis have been reported as rare side effects of COVID-19 vaccines, particularly with mRNA vaccines like Moderna and Pfizer.^21^ Diaz et al reported an incidence of vaccine-induced pericarditis of 1.8 cases per 100,000 individuals among the 2,000,287 people who received at least 1 dose of a COVID-19 vaccine. Thirty-seven cases of pericarditis were identified, with symptoms typically appearing 20 days after the most recent vaccination. Most cases were associated with the Pfizer/BioNTech vaccine (62.2%), followed by the Moderna vaccine (32.4%) and the Janssen/Johnson & Johnson vaccine (5.4%).^22^ Although evidence suggests that COVID-19 can cause pericarditis, our study showed that sometimes it led to overdiagnosis of pericarditis in patients with COVID-19 infection. Possible reasons include symptom overlap, atypical COVID-19 clinical presentation, and a diagnostic bias leading to attributing chest symptoms in COVID-19 to pericarditis, given the increased risk in this population. Moreover, our study period was toward the latter stages of the pandemic, when awareness of COVID-19 cardiac complications had improved but was still evolving, which may partly explain the high rate of misdiagnosis in these patients. Many patients were diagnosed with costochondritis or long-term COVID-19 afterward. Long-COVID has been associated with persistent chest pain and musculoskeletal pain.^23^ It would be interesting to further investigate the potential reasons for misdiagnosis in COVID-19 patients by comparing the rates and characteristics of misdiagnosed cases before and after the onset of the COVID-19 pandemic, as well as the latter stage of the pandemic.

Finally, our study sheds light on the importance of imaging and laboratory markers in establishing a diagnosis. More specifically, none of the patients with misdiagnosed pericarditis had pericardial enhancement on LGE-MRI, whereas most patients with accurately diagnosed pericarditis had pericardial enhancement. Current ESC guidelines include these markers as supporting evidence, but they are not a part of the diagnostic criteria. Other studies also showed the importance of pericardial enhancement. Klein et al^24^ state that cardiovascular magnetic resonance imaging (CMR) can help diagnose atypical presentations of acute pericarditis, particularly when other imaging modalities, such as echocardiograms, may be inconclusive. The predictive value of CMR in pericarditis was reaffirmed by Conte et al^25^ who found that patients with pericardial enhancement on LGE-MRI had a higher propensity to develop recurrent pericarditis. Due to its delineation and characterization of cardiac tissue, there is mounting support for the routine use of CMR for diagnosing and managing pericarditis.^26^ Al-Ani and Keeley add that CMR technology has advanced considerably. We can now use multiphase imaging relatively faster to accurately diagnose and manage pericarditis.^27^ Consequently, we may discern other pericardial syndromes presenting similarly to acute pericarditis, making CMR a high-yield diagnostic and prognostic first-line imaging modality. Our findings are timely, and the most recent International Position Statement on New Concepts and Advances in Multimodality Cardiac Imaging on Pericardial Disease^28^ as well as the ACC concise, clinical guidance document on pericarditis^29^ and the 2025 ESC Guidelines for the management of myocarditis and pericarditis^30^ highlights the importance of LGE-MRI, especially in complicated acute and recurrent pericarditis. Given the current high rates of misdiagnosed pericarditis and the critical need for more objective measures to assess these patients, the evaluation of pericardial enhancement on MRI could offer an objective, reproducible, and accurate tool for clinical practice.

### Study Limitations

Our study has many limitations. First, it is a descriptive study that sheds light on misdiagnosed pericarditis and is not powered to confirm significant differences. Future studies are needed to implement those findings in clinical practices prospectively. Second, due to its design, we cannot identify the specific health care providers who evaluated or directed the patients who were self-referred. Third, some patients in the misdiagnosed group needed an accurate diagnosis. Prospectively following these patients to better understand alternative diagnoses would be necessary. Fourth, some laboratory and EKG data were obtained from outside institutions; in recurrent or incessant cases, complete access to all historical tracings was not always possible. Finally, our study focused on patients referred with an outside diagnosis of pericarditis, allowing us to assess the rate of misdiagnosis.

## Conclusions

Diagnosing pericarditis is challenging. The prevalence of misdiagnosed pericarditis is 35%. Patients with misdiagnosed pericarditis had fewer cardiac comorbidities than patients with accurately diagnosed pericarditis. COVID-19 infection and the incidental discovery of pericardial effusion are associated with misdiagnosis. Inflammatory markers and LGE-MRI enhancement can help refine diagnosis and should be incorporated into future diagnostic criteria definitions.

## Funding support and author disclosures

Dr Klein receives research grants from 10.13039/100016492Kiniksa, Cardiol Therapeutics, and Ventyx Scientific; and serves on the advisory Boards of Kiniksa, Cardiol Therapeutics, and Ventyx Scientific. All other authors have reported that they have no relationships relevant to the contents of this paper to disclose.

## References

[1] Adler Y., Charron P., Imazio M. (2015). 2015 ESC guidelines for the diagnosis and management of pericardial diseases: the task force for the diagnosis and management of pericardial diseases of the european society of cardiology (ESC)Endorsed by: the european association for cardio-thoracic surgery (EACTS). Eur Heart J.

[2] Chiabrando J.G., Bonaventura A., Vecchié A. (2020). Management of acute and recurrent pericarditis: JACC state-of-the-art review. J Am Coll Cardiol.

[3] Gouriet F., Levy P.-Y., Casalta J.-P. (2015). Etiology of pericarditis in a prospective cohort of 1162 cases. Am J Med.

[4] Imazio M., Hoit B.D. (2013). Post-cardiac injury syndromes. An emerging cause of pericardial diseases. Int J Cardiol.

[5] Agrawal A., Berglund F., Kumar A.K. (2023). Adverse prognosis in patients with postcardiac injury syndrome-related recurrent pericarditis: effect of gender. JACC Adv.

[6] Yadav R., Satti D.I., Malwankar J. (2024). Pericarditis after catheter ablation for atrial fibrillation: predictors and outcomes. JACC Clin Electrophysiol.

[7] Nakhla S., Mentias A., Rymer C. (2022). Acute pericarditis after atrial fibrillation ablation: incidence, characteristics, and risk factors. Heart Rhythm O2.

[8] Lehto J., Gunn J., Björn R. (2020). Adverse events and survival with postpericardiotomy syndrome after surgical aortic valve replacement. J Thorac Cardiovasc Surg.

[9] Alachkar M.N., Lehrke M., Marx N., Almalla M. (2020). Post-cardiac injury syndrome after transcatheter mitral valve repair using MitraClip system: a case report. Eur Heart J Case Rep.

[10] Thadani U., Chopra M.P., Aber C.P., Portal R.W. (1971). Pericarditis after acute myocardial infarction. Br Med J.

[11] Figueras J., Juncal A., Carballo J., Cortadellas J., Soler J.S. (2002). Nature and progression of pericardial effusion in patients with a first myocardial infarction: relationship to age and free wall rupture. Am Heart J.

[12] Verma B.R., Montane B., Chetrit M. (2020). Pericarditis and post-cardiac injury syndrome as a sequelae of acute myocardial infarction. Curr Cardiol Rep.

[13] Oliva P.B., Hammill S.C., Talano J.V. (1994). Effect of definition on incidence of postinfarction pericarditis. It is time to redefine postinfarction pericarditis?. Circulation.

[14] Imazio M., Negro A., Belli R. (2009). Frequency and prognostic significance of pericarditis following acute myocardial infarction treated by primary percutaneous coronary intervention. Am J Cardiol.

[15] Tung-Chen Y. (2020). Acute pericarditis due to COVID-19 infection: an underdiagnosed disease?. Med Clin (Engl Ed).

[16] Campos I.D., Salgado A., Azevedo P., Vieira C. (2019). Dressler's syndrome: are we underdiagnosing what we think to be rare?. BMJ Case Rep.

[17] Hoffmann M., Kleine-Weber H., Schroeder S. (2020). SARS-CoV-2 cell entry depends on ACE2 and TMPRSS2 and is blocked by a clinically proven protease inhibitor. Cell.

[18] Halushka M.K., Vander Heide R.S. (2021). Myocarditis is rare in COVID-19 autopsies: cardiovascular findings across 277 postmortem examinations. Cardiovasc Pathol.

[19] Kytö V., Sipilä J., Rautava P. (2014). Clinical profile and influences on outcomes in patients hospitalized for acute pericarditis. Circulation.

[20] Boehmer T.K., Kompaniyets L., Lavery A.M. (2021). Association between COVID-19 and myocarditis using hospital-based administrative data - United States, March 2020–January 2021. MMWR Morb Mortal Wkly Rep..

[21] Straus W., Urdaneta V., Esposito D.B. (2022). Analysis of myocarditis among 252 million mRNA-1273 recipients worldwide. Clin Infect Dis.

[22] Diaz G.A., Parsons G.T., Gering S.K., Meier A.R., Hutchinson I.V., Robicsek A. (2021). Myocarditis and pericarditis after vaccination for COVID-19. JAMA.

[23] Salatzki J., Ochs A., Weberling L.D. (2024). Absence of cardiac impairment in patients after SARS-CoV-2 infection: a long-term follow-up study. J Cardiovasc Magn Reson.

[24] Klein A.L., Abbara S., Agler D.A. (2013). American society of echocardiography clinical recommendations for multimodality cardiovascular imaging of patients with pericardial disease: endorsed by the society for cardiovascular magnetic resonance and society of cardiovascular computed tomography. J Am Soc Echocardiogr.

[25] Conte E., Agalbato C., Lauri G. (2022). Cardiac MRI after first episode of acute pericarditis: a pilot study for better identification of high risk patients. Int J Cardiol.

[26] Ho N., Nesbitt G., Hanneman K., Thavendiranathan P. (2021). Assessment of pericardial disease with cardiovascular MRI. Heart Fail Clin.

[27] Al-Ani M., Keeley E.C. (2022). Emerging role of cardiac MRI in acute pericarditis. Int J Cardiol.

[28] Klein A.L., Wang T.K.M., Cremer P.C. (2024). Pericardial diseases: international position statement on new concepts and advances in multimodality cardiac imaging. JACC Cardiovasc Imaging.

[29] Wang T.K.M., Klein A.L., Cremer P.C. (2025). 2025 concise clinical guidance: an ACC expert consensus statement on the diagnosis and management of pericarditis: a report of the American College of Cardiology Solution Set Oversight Committee. J Am Coll Cardiol.

[30] Schulz-Menger J., Collini V., Gröschel J. (2025). ESC Scientific Document Group. 2025 ESC guidelines for the management of myocarditis and pericarditis. Eur Heart J..

